# Cross-Cohort Microbiome Analysis of Salivary Biomarkers in Patients With Type 2 Diabetes Mellitus

**DOI:** 10.3389/fcimb.2022.816526

**Published:** 2022-01-25

**Authors:** Chuqi Gao, Ying Guo, Feng Chen

**Affiliations:** ^1^ Central Laboratory, Peking University School and Hospital of Stomatology, Beijing, China; ^2^ Department of Stomatology, General Hospital of Shenzhen University, Shenzhen, China

**Keywords:** type 2 diabetes mellitus, biomarker, human microbiome, sequence analysis, meta-analysis, 16S

## Abstract

Several studies have ascertained differences in salivary microbiota between patients with type 2 diabetes mellitus (T2DM) and healthy populations. However, the predictive accuracy and reproducibility of these 16S rRNA sequencing analyses when applied to other cohorts remain enigmatic. A comprehensive analysis was conducted on the included 470 samples from five researches in publicly available databases. The discrepancy and predictive accuracy of salivary microbiota between T2DM patients and healthy populations were evaluated from multiple perspectives, followed by the identification of salivary biomarkers for DM. Next, a classification model (areas under the curves = 0.92) was developed based on a large sample. The model could be used for clinical diagnosis and prognostic monitoring and as a basis for hypothesis-driven mechanistic researches. Furthermore, the research heterogeneity across geographic regions suggested that microbiological markers might not become a uniform clinical standard in human beings. They rather identify abnormal alterations under the microbiological characteristics of a specific population.

## Introduction

Type 2 diabetes mellitus (T2DM), the most prevalent type of diabetes mellitus (DM), is attributed to a progressive decrease in insulin secretion and insulin resistance. It is ultimately characterized by poor glucose tolerance, hyperglycemia, and overt DM, accounting for 90-95% of DM population (Alvarenga et al., 2020). The International Diabetes Federation estimates that the prevalence of T2DM in the global adult population will exceed 10% by 2040 (Ogurtsova et al., 2017). There exist multiple diagnostic methods of DM, including fasting serum/plasma glucose tests, oral glucose tolerance tests, and interim glucose tests combined with clinical symptoms. HbA1c has been proposed as a screening and diagnostic test for DM (Higgins, 2013). However, these methods are invasive, which limits the possibility of large-scale screening. Thus, there is still an urgent need to explore easy, non-invasive, and highly accurate screening methods.

Periodontal disease is one of the common complications of DM, which has drawn attention to the oral microbiology of T2DM patients, expecting to find non-invasive biomarkers specific to DM in the oral cavity (Kocher et al., 2000). Previously, several researches have reported that specific periodontal microbes are associated with DM and that the alterations in the periodontal microbial community are potential precursors to periodontal diseases (Long et al., 2017; Shi et al., 2020; Omori et al., 2021). DM has been documented to reduce the diversity and community stability of oral microorganisms (Sabharwal et al., 2019; Yang et al., 2020). However, there also exist multiple opposite conclusions (Casarin et al., 2013). Several studies have revealed salivary biomarkers and predictive models for T2DM (Sun et al., 2020; Liu et al., 2021). However, the predictive accuracy and reproducibility of these biomarkers and models remain poorly identified when applied to other cohorts. In conclusion, it is generally accepted that the changes in the oral microbiology are correlated with the pathogenesis of T2DM, but there has never been a consensus on the specific pathogenic microorganisms.

It is urgent to validate the associations of the human oral microbiome and DM across populations, geographic regions, and cohorts. Large-scale cross-cohort researches combine and analyze raw sequencing data from massive samples. They provide a powerful and bias-reducing method to decrease the impact of confounding factors such as epidemiological characteristics and operative techniques, realizing the uniformity of results across multiple studies worldwide. Therefore, these researches have effects comparable to multi-center large-sample studies (Thomas et al., 2019). Although microbiological researches of T2DM and periodontitis are of great interest, there have not been any large cross-cohort studies to date.

As oral microbiology has been increasingly studied, there is a research observing that the sample collection method can significantly impact the results of oral microbiome analyses (Yano et al., 2020). Traditionally, the oral microbiome in periodontal disease has been characterized by sampling subgingival plaque (Abusleme et al., 2013). However, more researches have chosen to collect saliva samples to characterize the oral microbiome due to the easy sampling. In addition to sampling, the selection of the hypervariable regions in the sequenced 16S rRNA gene has an impact on characterizing the diversity of the oral microbiome (Griffen et al., 2012). The primer pairs spanning the V3-V4 hypervariable region captured better diversity in contrast to primer pairs spanning the V1-V3 region (Castelino et al., 2017). Illumina platform is the most commonly used sequencing platform in second-generation sequencing (Pichler et al., 2018).

This research harvested 470 samples from five studies in publicly available databases, where DNA was extracted from saliva samples to amplify V3-V4 hypervariable regions in the 16S rRNA gene and conduct sequencing on the Illumina platform. A comprehensive analysis was implemented to evaluate the salivary microbial discrepancy and predictive accuracy between T2DM patients and healthy populations. Then, salivary biomarkers for T2DM were predicted and a classification model was constructed based on large-scale samples.

## Materials and Methods

### Public Data Collection

The sequencing raw data of 16S rRNA of T2DM patients and healthy controls were harvested from published studies on PubMed and Embase with the inclusion of all publication dates and all languages. Analyses of this research were conducted on T2DM patients who met the inclusion criteria, and the complete oral microbiome was evaluated using 16S rRNA sequencing technology.

The inclusion criteria were as follows: (1) case-control or cross-sectional studies, or the researches published as original articles; (2) independent studies, or the most recent or informative reported results in the case of multiple reports for the same group or subgroup; (3) all samples collected as unstimulated saliva; (4) 16S rRNA sequencing using the Illumina platform, amplification of V3-V4 hypervariable region in the 16S rRNA gene; (5) the studies providing raw data of 16S rRNA sequencing for all samples. Reviews, letters to the editor, monographs, conference papers, book chapters, case reports, unpublished data, and animal studies were excluded. Also, researches were excluded if at least one of the following criteria was present: (1) the studies without a non-diabetic control group; (2) the patient with a concurrent systemic disease other than T2DM or undergoing treatment such as implant placement, crown orthodontics, or periodontal surgery; (3) the primary finding not related to T2DM.

After screening, only seven studies fully met the inclusion criteria, among which only four submitted the raw data in the Sequence Read Archive (SRA) database of National Center for Biotechnology Information (NCBI). We sent e-mails requesting raw data to the corresponding authors of the other three studies but only received data returned by Dr. Amarpreet Sabharwal. Therefore, this work included only five studies with accessible sample metadata and high-throughput sequencing performance for the V3-V4 region of the 16S rRNA gene. The raw data for four of the five studies and the independent cohort were read and downloaded from the SRA database of NCBI using the SRA Toolkit (V.2.9.2) with the following sequence numbers: PRJNA561495 by Yang et al., PRJNA601054 by Sun et al., PRJNA609009 by Liu et al., PRJNA679485 by Almeida-Santos et al, and the independent cohort with the number of PRJNA664107. The readers can download them by https://www.ncbi.nlm.nih.gov/sra/?term=PRJNA561495, https://www.ncbi.nlm.nih.gov/sra/?term=PRJNA601054, https://www.ncbi.nlm.nih.gov/sra/?term=PRJNA609009, https://www.ncbi.nlm.nih.gov/sra/?term=PRJNA679485 and https://www.ncbi.nlm.nih.gov/sra/?term=PRJNA664107.

### Data Pre-Processing

The results were stored in FASTQ (referred to as fq) format file, which contained sequence information of reads and their corresponding sequencing quality information. Raw reads were firstly filtered by Trimmomatic v0.33. Then the primer sequences were identified and removed by cutadapt 1.9.1, which finally generated high-quality reads without primer sequences. Based on overlapping sequences, high-quality reads were assembled by FLASH v1.2.7, which generated clean reads. Chimeric sequences were identified and removed by UCHIME v4.2, generating effective reads.

### Quality Assessment of Sequencing Data

After processing the raw data, data quality was estimated based on parameters, such as read length, counts of reads at each stage, guanine-cytosine (GC) content, PHRED quality score threshold of 20 (Q20) and Q30 quality, and effective values. All samples had sufficient sequencing depth, except for three samples in Almeida-Santos’s study. The end of the rarefaction curves showed a gentle rise, indicating that sequencing saturation was achieved for all samples and that operational taxonomic units (OTUs) covered most of the microbial species present in saliva (see **Supplementary Figure 1**).

### Data Annotation and Statistical Analysis

Usearch was applied to cluster reads with similarity above 97.0%, generating OTUs (Edgar, 2013). Taxonomic annotations of feature sequences were processed by a Bayesian classifier using SILVA as a reference database (Sierra et al., 2020). Alpha and beta diversity metrics were evaluated by QIIME2 (Fung et al., 2021). In identifying T2DM versus healthy controls, the Wilcoxon rank sum test was used to determine statistical differences between groups, considering that there were only two groups which did not follow a normal distribution. Additionally, in identifying study heterogeneity among five groups, Anosim analysis was used. The randomForest in R package was applied to construct a random forest (RF) model and calculate the effect of each variable on the heterogeneity of observations at each node of the classification tree to obtain MeanDecreaseGini values. Then a 10-fold cross-validation was performed by dividing the dataset into ten parts and experimenting with nine of them in turn as the training set and one as the test set. The RF model was reconstructed using the one with the highest accuracy. The test set was trained again. Next, receiver operating characteristic (ROC) curves were plotted using the output predicted values, followed by the calculation of area under the ROC curve (AUC) values, accuracy, precision, and recall. FAPROTAX database was utilized to perform species annotation on feature sequences based on reference phylogenetic tree. Potential functions and functional genes in samples were predicted, which further revealed the difference in functions between samples or groups. The significance of difference in function abundance between samples was evaluated by G-test (the number of annotated functional genes > 20) and Fisher (the number of annotated functional genes < 20) in STAMP.

## Results

### The Characteristics of the Large Scale Dataset

In this research, the sequencing raw data of 16S rRNA from five studies were investigated to assess differences of salivary microbiome between T2DM patients and healthy populations and to identify DM-specific biomarkers. In total, 273 samples were obtained from T2DM patients and 200 samples were collected from healthy controls. Demographic information is presented in **Table 1**, including age, sex, body mass index (BMI), and country of the subjects in each study. All samples were sequenced at sufficient depth except for 3 samples (SRR13084941, SRR13084942, and SRR13084945) from the research by Almeida-Santos et al. These samples were excluded for further analysis. A total of 21,995,091 paired-end (PE) reads were generated from the final 470 samples. After that, 17,894,743 clean reads were obtained after the quality control and assembly of the PE reads. The total number and the average number of reads per study are also recorded in **Table 1**. An average of 38,074 clean reads was generated per sample. Quality control was performed on the raw data, including the removal of the low-quality reads, the filtration based on length, and the generation of the high-quality reads. Consistent processing was conducted for all raw sequencing data on the Quantitative Insights Into Microbial Ecology platform.

**Table 1:** Clinical Characteristics of Large-Scale Dataset^*^.

	Group (N)	Age (average ± s.d)	Sex (F/M)	BMI (average ± s.d)	Total Reads	Average Reads	Country
**US**	DM (79)	52.99 ± 8.53	36/43	32.97 ± 8.12	5582131	39036	USA
	Control (64)	39.73 ± 14.36	40/24	27.73 ± 5.80			
**Shandong**	DM (70)	54.63 ± 12.13	20/50	26.63 ± 4.64	3593179	35227	China
	Control (32)	49.19 ± 8.72	16/16	24.77 ± 2.70			
**Anhui**	DM (75)	58.56 ± 10.46	43/32	25.67 ± 3.48	5186681	38998	China
	Control (58)	37.21 ± 13.87	41/17	22.22 ± 3.05			
**Sichuan**	DM (24)	47 (33−65)	11/13	25.97 ± 2.32	1652946	36732	China
	Control (21)	47.24 (35-61)	11/10	23.23 ± 2.08			
**Portugal**	DM (25)	62.72 ± 7.12	8/17	28.36 ± 5.20	1879806	39996	Portugal
	Control (22)	59.91 ± 8.88	6/16	26.79 ± 4.52			

*The V3-V4 hypervariable region in the 16S rRNA gene was amplified in all studies.

### The Identification of the Heterogeneity in the Potential Studies

**Figure 1:**
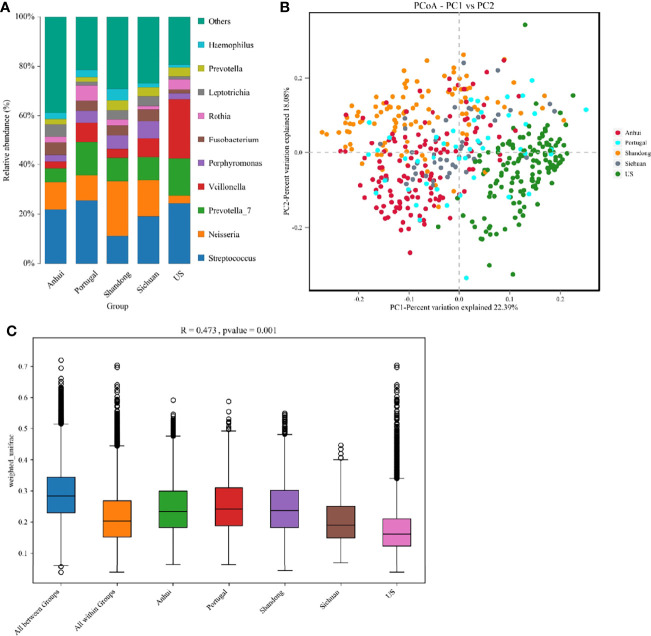
**(A)** The structure analysis of the microbial community. The composition statistics were calculated for each sample at the phylum, order, family, genus, and species levels. This figure showed bar graphs of microbial abundance at the genus level for the five studies. **(B)** The PCoA of all samples from the five studies based on Weighted-Unifrac distances. X-axis and y-axis represented two eigenvalues that maximized the differences between samples, respectively. **(C)** R and *p* values for beta diversity based on Weighted-Unifrac distances calculated using the Anosim analysis (analysis of Similarities). The closer the R value was to 1, the greater the differences between groups were than the differences within groups; the smaller the R value, the less significant the differences between the groups. *p* < 0.05 showed high reliability of the test. The box above “All between Groups” indicated the Weighted-Unifrac distance data of the samples among all groups, while the box above “All within Groups” indicated the Weighted-Unifrac distance data of the samples within all groups. The box below represented the Weighted-Unifrac distance data of samples within different groups.

The heterogeneity of the potential studies was explored due to the technical and biological differences among these studies. From **Figures 1A, B**, it was seen that there were significant differences in the microbial species contained in the five researches, which was tentatively speculated to be related to their geographical discrepancies. A typical phenomenon in **Figure 1B** was that the distribution of the characteristics of the three studies in Shandong, Anhui, and Sichuan was concentrated in quadrants 1, 2, and 4, whilst the study in the USA was concentrated in quadrant 3 and the study in Portugal was distributed in all the quadrants. It was thus speculated that microbial differences might also be influenced by ethnicity.

In the principal coordinate analysis (PCoA), there was no corresponding statistical test to conclude whether the differences between the different groups were significant or not. Therefore, the significance of the differences was calculated using the Anosim analysis (**Figure 1C**), in which R = 0.473 indicated significant differences between groups in the five studies (*p* = 0.001).

### Salivary Microbial Differences Between T2DM Patients and Healthy Controls

A total of 197 species in 148 genera from 13 phyla, 20 orders, and 43 families were detected. There existed no significant differences in the salivary microbial community between T2DM patients and healthy controls from all aspects assessed. Alpha diversity analysis manifested no significant differences between the two groups in terms of mean Shannon, Simpson, Abundance-based Coverage Estimators (ACE), Chao1, and Phylogenetic diversity (PD) whole tree indexes (**Table 2**).

**Table 2:** Alpha Diversity Indicators.

	Control	DM	p value
**Shannon**	4.7722 ± 0.0587	4.817 ± 0.0407	0.5179
**Simpson**	0.9013 ± 0.0064	0.9124 ± 0.0028	0.0844
**ACE**	232.1372 ± 4.7197	233.271 ± 4.5195	0.8649
**Chao1**	233.602 ± 4.9316	235.2331 ± 4.6347	0.8127
**PD whole tree**	14.5491 ± 0.3559	14.407 ± 0.3075	0.7632

**Figure 2:**
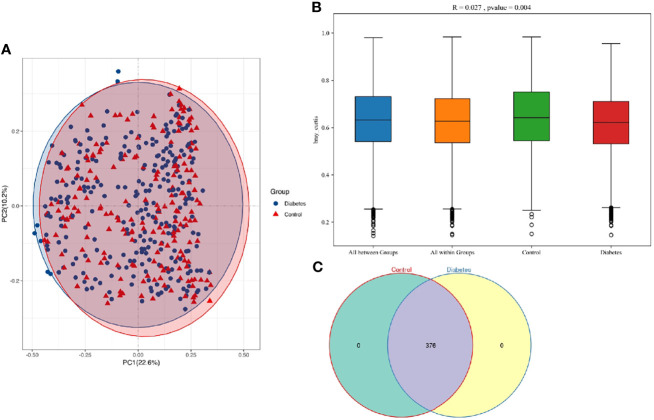
**(A)** PCoA based on Bray-Curtis distances for all samples from T2DM patients and healthy controls (T2DM, n = 273; control, n = 197). Ellipses represented 95% confidence level. The blue and red ellipses almost overlapped, indicating insignificant differences between T2DM patients and healthy populations. **(B)** R and *p* values for the beta diversity based on Bray-Curtis distance calculated using Anosim analysis (analysis of Similarities). The closer the R value was to 1, the greater the differences between groups than the differences within groups. The smaller the R value was, the less significant the differences between them. *p* < 0.05 showed the high reliability of the test. **(C)** The numbers in each independent or overlapping region of the Venn diagram representing the number of features in each corresponding set, indicating that the “core microbiome” of T2DM patients and healthy controls overlapped completely.

In the analysis of the beta diversity, the PCoA revealed that the saliva samples from T2DM and control groups could not be separated, suggesting insignificantly different salivary microorganisms. R = 0.027 from the Anosim analysis further verified insignificant difference between groups (*p* = 0.004) (**Figures 2A, B**). Venn diagram indicated that T2DM patients shared the same salivary “core microbiome” as the healthy populations and that the salivary microbiota of T2DM patients might not have specific characteristics compared to the control individuals (**Figure 2C**). The fully overlapping “core microbiome” also supported the further analysis of the related microbes between the two groups at the phylum, genus, and species levels.

**Figure 3:**
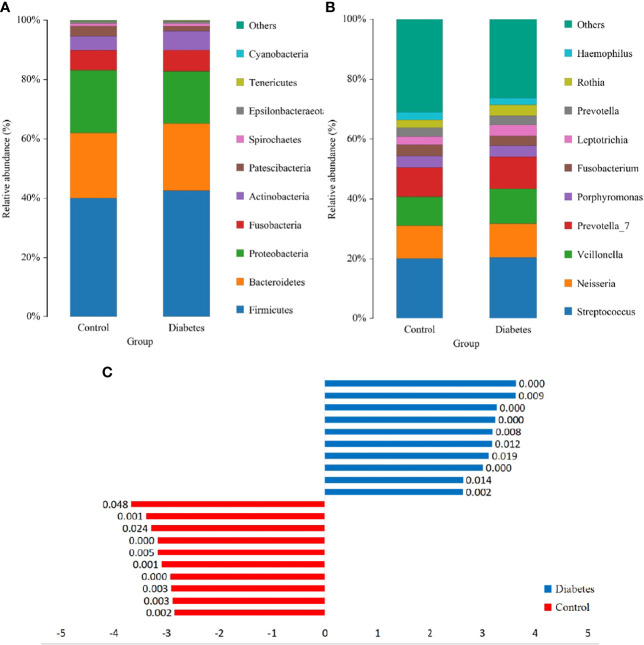
The structure analysis of the microbial community. **(A)** and **(B)** are the bar graphs of microbial abundance at the phylum and genus levels, respectively. **(C)** LDA bar graph. Blue and red bars represented LDA values for taxa enriched in the T2DM group and those enriched in the healthy controls with *p*-values labeled next to the bars, respectively.

The Wilcoxon rank-sum test was utilized to analyze differences in salivary microorganisms between groups from the phylum to the OTU level. At the phylum level, the salivary microbiota of T2DM patients and healthy controls was dominated by p. Firmicutes (41.74% and 39.76%), followed by p. Bacteroidetes (23.10% and 22.08%), p. Proteobacteria (17.97% and 21.31%), p. Fusobacteria (7.11% and 6.77%), and p. Actinobacteria (6.38% and 4.88%), accounting for approximately 95% of the total bacteria (**Figure 3A**). The increase of the p. Actinobacteria in T2DM patients was significant (*p* = 0.001), which was similar to the findings of Yang et al. and Long et al. (Long et al., 2017; Yang et al., 2020). We also found an elevation in the ratio of p. Firmicutes/p. Bacteroidetes (1.181 and 1.180), although this change was not significant (*p* > 0.05). The ratio of p. Firmicutes/p. Bacteroidetes has been documented to enhance in the gut of T2DM patients and be associated with the mild inflammation and the improved capacity of obtaining energy from food (Pascale et al., 2019). There is also a large-sample oral research that confirms the enhancement of this ratio in the oral cavity of T2DM patients (Chen et al., 2020).

At the genus level, the dominant genera were Streptococcus (20.41% and 20.28%), Neisseria (11.47% and 10.94%), Veillonella (11.28% and 9.53%), Prevotella_7 (10.91% and 9.82%), Porphyromonas (3.95% and 3.92%), and Rothia (3.63% and 2.70%) (**Figure 3B**), among which only the augmentation in Rothia was significant (*p* < 0.001) in T2DM patients.

The significant differences in species abundance between the two groups were analyzed at the species level. Only uncultured_bacterium_g_Rothia among the top 15 species in abundance augmented obviously [*p* = 2.0 × 10^(-11)] in the T2DM patients. Besides, Prevotella_7, Veillonella, and a group of uncultured Lactobacillus elevated comparatively significantly (*p* < 0.05). The remaining species did not significantly differ between the two groups (**Supplementary Table 1**). Among the five researches, the subjects from Portugal and US had higher levels of Rothia (**Figure 1A**). To exclude the possibility that one study had a disproportionate effect on the results, these two studies were removed separately, which displayed that the elevation of Rothia remained significant [*p* = 6.2 × 10^(-10) and *p* = 2.5 × 10^(-5)]. This result illustrated the general elevation of Rothia in the T2DM population. To exclude confounding factors, the separate regression analyses were implemented for the content of Rothia according to known sex, age, BMI, and smoking frequency, which exhibited insignificant linear relationship (R < 0.05).

To further dissect the presence of significantly different bacteria between T2DM patients and control individuals, a linear discriminant analysis (LDA) Effect Size (LEfSe) analysis was performed from the phylum to the OTU level. The Kruskal-Wallis rank-sum test was conducted for OTUs with LDA scores > 2, which depicted a significant difference (*p* < 0.05) in some OTUs between T2DM patients and healthy controls (**Figure 3C**).

### The Microbial Classification Model for the Saliva With T2DM

**Figure 4:**
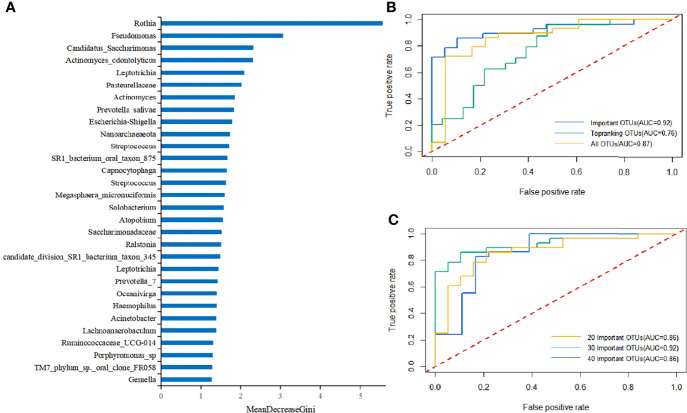
**(A)** Key components of the RF model constructed using all OTUs to distinguish differences between T2DM patients and healthy controls (MeanDecreaseGini values represented the importance of species in the RF model). **(B)** The 10-fold cross-validation was performed on the RF model, and the model was reconstructed using the sample with the highest precision. The ROC curves and AUC values of the overall test set are shown above. The top 30 important and top-ranking OTUs were selected, respectively, where each OTU could be considered as an independent species. **(C)** The 10-fold cross-validation was performed on the RF model, and the model was reconstructed using the sample with the highest accuracy. The ROC curves and AUC values of the overall test set are shown above. 20 Important OTUs, 30 Important OTUs, and 40 Important OTUs represented the top 20, 30, and 40 OTUs in importance, respectively, where each OTU could be considered as an independent species.

An RF model was firstly constructed using all OTUs. Then, we evaluated the impacts of each variable on the heterogeneity of the observations at each node of the classification tree and measured the importance of the variables by MeanDecreaseGini to obtain the top 20 key OTUs (**Figure 4A**). Among them, Rothia sp. was the most key one, which was consistent with the results of the beta diversity analysis. The next most key factors were Pseudomonas, Candidatus_Saccharimonas, Actinomyces_odontolyticus, Leptotrichia, Pasteurellaceae, Actinomyces, Prevotella_salivae, Escherichia-Shigella, and Nanoarchaeaeota.

Further, a T2DM ancillary diagnostic model was developed. The RF models were constructed based on the top 30 most important and richest OTUs and all OTUs, respectively, and were tested with 10-fold cross-validation based on OTUs. The comparison of the obtained ROC curves and AUC values revealed that the top 30 most important OTUs had higher AUC values (**Figure 4B**), which was consistent with the findings of previous studies. In addition, to determine the number of factors included in the model, the RF models containing the top 20, 30, and 40 OTUs in importance were constructed, and the obtained ROC curves and AUC values were compared. The findings demonstrated that the top 30 OTUs in importance had higher AUC values (**Figure 4C**). It was worth mentioning that the RF model was constructed using only one variable, Rothia sp., which manifested that the AUC value was still as high as 0.69. To our knowledge, no OTU has ever had such a high AUC value as an independent indicator, which again proves the importance of Rothia in the diagnosis of T2DM.

Therefore, the RF model containing the top 30 OTUs in importance were finally identified as the T2DM ancillary diagnostic model (**Supplementary Table 2**). Rothia sp. had the highest IncNodePurity value, indicating its irreplaceable importance in the model (AUC = 0.92, accuracy = 0.83, precision = 0.83, and recall = 0.89).

### Inter-Study Transfer Validation of a Salivary Microbial Classification Model

**Figure 5:**
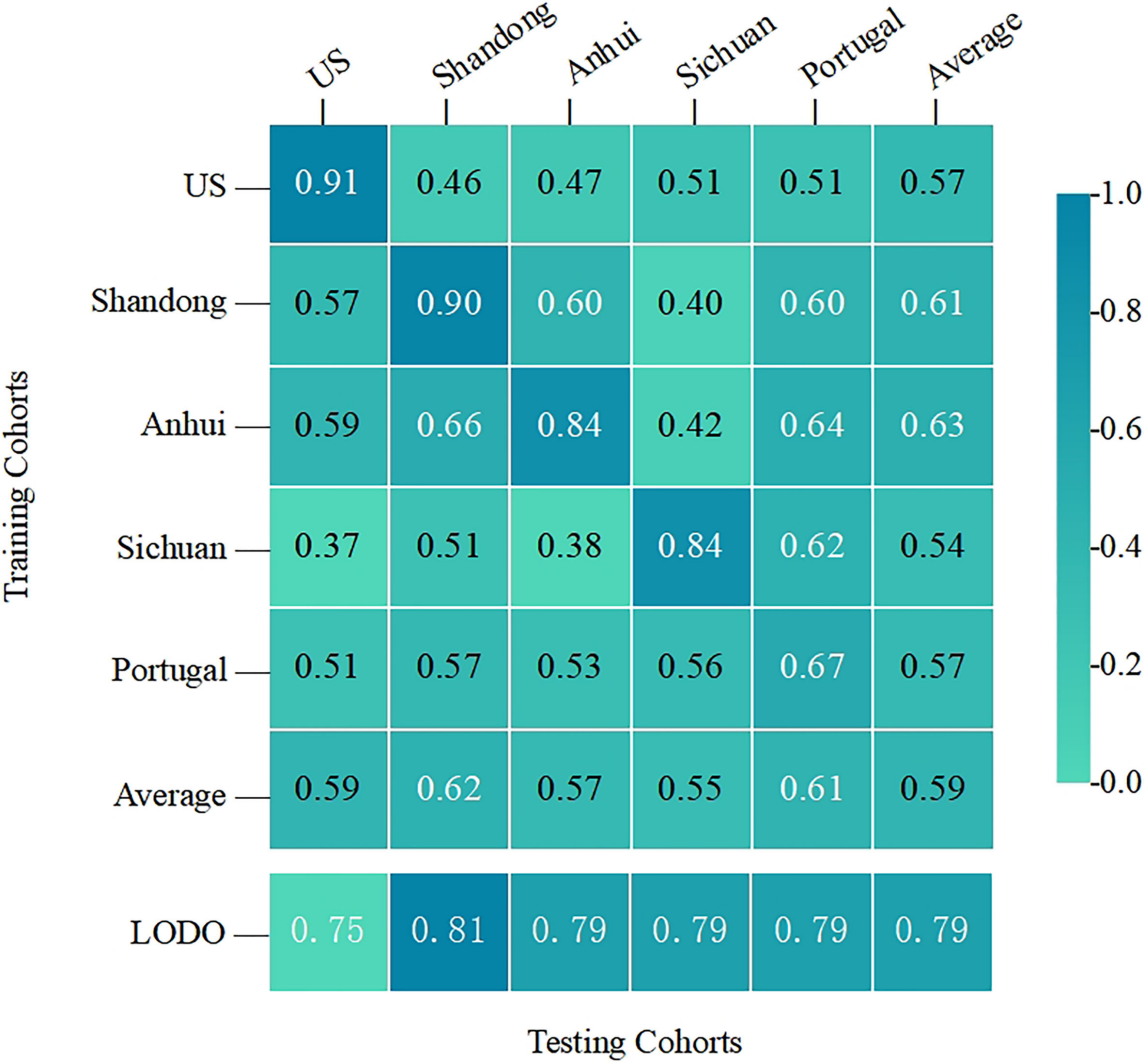
The cross-prediction matrix thoroughly showed the AUC values of the five studies themselves and between them for the prediction of T2DM. The values on the diagonal were the results of the cross-cohort validation within each study. The non-diagonal AUC values were obtained by training the classifier on the study in each row and tested on the study in the corresponding column.

To test whether the top 30 important OTUs identified were generalizable and robust across multiple studies, leave-one-dataset-out (LODO) validation and inter-study transfer validation were performed on the entire sample (**Figure 5**). The mean LODO was 0.79, demonstrating that the conclusion was general across the five studies with negligible influence of any single study. The AUC values for the inter-study transfer validation ranged from 0.37 to 0.91 with a wide span and a mean value of 0.59. The values on the diagonal were high enough, the highest of which was close to the AUC value of the RF model. This indicated that the important features identified by the RF model had good diagnostic strength when T2DM patients had similar clinical characteristics with the healthy population, such as the same geographic region. However, the lower non-diagonal values suggested that the cross-validation within each study was generally better than that between studies. These results provided some evidence that a range of clinical characteristics represented by geographic region could severely afflict the diagnostic ability of the RF model for T2DM.

### Altered Salivary Microbial Function in T2DM Patients

Currently, the Greengene database is not updated as fast as SILVA, so we used the SILVA as the reference database. However, both the commonly used Phylogenetic Investigation of Communities by Reconstruction of Unobserved States (PICRUSt) and BugBase analyses are only applicable to the Greengene database. Therefore, FAPROTAX database was applied for functional annotation prediction of all the samples, which has better prediction accuracy but may have less prediction coverage compared to PICRUSt. Human pathogen septicemia was only one significant functional gene alteration in the salivary microbiota of T2DM patients observed (*p* < 0.01) (**Supplementary Figure 2**). This evidence suggested an association between T2DM and septicemia. Human pathogens septicemia was added for reconstructing the RF model as the 31st variable, yielding an AUC value of 0.85. However, we unexpectedly found that with 31 variables, the recall improved from 0.89 to 0.93, which may be more favorable for future applications in large-scale screening. No other significant alteration had been observed in the salivary microbiota of T2DM patients.

### Validation in an Independent Cohort of Subgingival Samples

46 subgingival periodontal samples with the sequence number PRJNA664107 (Diabetes n=15, Control=31) were selected as an independent cohort for validation and obtained an accuracy rate of 0.78. This rate is comparable to the accuracy of the inter-study validation, which indicates that the constructed model has good predictive power both in the included studies and outside of them.

## Discussion

By combining raw data from available datasets for the unified analysis, our two major findings were as follows: first, salivary microbial diversity was not significantly different between T2DM patients and healthy populations, which was confirmed by multiple statistical means. The second finding was that Rothia sp. was significantly higher in T2DM patients than in healthy population [*p* = 2.0 × 10^(-11)], which was the joint result of the Anosim analysis, the Wilcoxon rank-sum test, the Kruskal-Wallis rank-sum test, and the RF model significant factor analysis with the exclusion of the effects of every single study. Therefore, we concluded that Rothia sp. was the most representative salivary biomarker in T2DM patients. It was worth mentioning that a significant elevation of Rothia sp. was observed in three of five included studies (*p* < 0.001), but was not specifically mentioned in the text (Sabharwal et al., 2019; Sun et al., 2020; Yang et al., 2020).

Rothia sp. belongs to p. Actinomycetes, the increase of which was also confirmed in T2DM patients. In fact, the association of p. Actinomycetes with T2DM has attracted increasing attention, but there is no consensus whether it increases or decreases in T2DM (Long et al., 2017; Matsha et al., 2020). Rothia sp. is a popular nitrate-reducing bacterium in the oral cavity and participates in the nitrate (NO3-)-nitrite (NO2-)-nitric oxide (NO) pathway, the positive impacts of which on NO activity favor the cardiovascular diseases (Vanhatalo et al., 2018). However, the discoveries of the present study suggested that this positive effect did not seem to apply to DM and that the exact role of nitrogen metabolism in the pathogenesis of DM remained to be further investigated. Prevotella sp., the next most critical biomarker of T2DM identified in this study, has been reported to be a pathogenic genus associated with insulin resistance and poor glucose tolerance (Pedersen et al., 2016).

However, the shift from a single pathogen doctrine to a microecological doctrine about inflammation and dysbiosis suggests that we should focus more on the whole picture of the flora rather than on some specific pathogen. Although Rothia sp. is of great significance in T2DM diagnosis as a common feature of T2DM patients in all geographical populations, it is still necessary to find an appropriate complementary diagnostic model to improve clinical diagnosis. Therefore, another crucial result of our work was the construction of a highly accurate T2DM prediction model based on the large sample with an AUC of 0.92, which could be applied for clinical diagnosis and prognostic monitoring.

Of the five studies included, two studies found decreased microbial alpha diversity in the saliva of T2DM patients (Sabharwal et al., 2019; Yang et al., 2020), whereas three studies observed insignificant changes (Sun et al., 2020; Almeida-Santos et al., 2021; Liu et al., 2021). However, there also exist unincluded researches elucidating that alpha diversity is elevated in T2DM patients (Casarin et al., 2013). Sun et al. and Almeida-Santos et al. also noted that the composition of the salivary microbial community in T2DM patients with periodontitis converged to that of healthy individuals after effective glycemic control (Sun et al., 2020; Almeida-Santos et al., 2021). However, the research of Yang et al. elucidated that the diversity of the salivary microbial community did not change obviously after metformin or combination therapy, which meant that treatment might not lead to flora recovery (Yang et al., 2020). In terms of the beta diversity between T2DM patients and healthy controls, three studies concluded that the differences were significant (Sabharwal et al., 2019; Sun et al., 2020; Yang et al., 2020). For example, the PCoA of Unweighted-UniFrac distance elaborated that the salivary microbiota distribution was more dispersed in non-diabetic individuals than in individuals with a history of T2DM (Yang et al., 2020). Additionally, two other studies uncovered similar distributions between groups (Almeida-Santos et al., 2021; Liu et al., 2021). However, in the present research, the differences once observed were practically offset after expanding the sample size. These contradictory results ultimately point to the conclusion that T2DM and healthy population have a similar salivary microbial composition.

Although the oral microbiome exhibited little difference in microbial diversity between T2DM patients and healthy controls, several biomarker differences were significant at each taxonomic level and these biomarkers were validated to be prevalent across the five studies, such as Rothia, on which we focused in our analysis. We found that there was indeed a difference in salivary microbial composition between T2DM patients and healthy populations, specifically in terms of biomarker content, but not diversity. It is clear that diversity is not sensitive enough in characterizing salivary microorganisms. Although Rothia as a single biomarker was valid to demonstrate differences between T2DM patients and healthy populations with a high prediction accuracy of 0.69, it was not high enough. Therefore, we attempted to construct a model with more variables in unison using the top 30 significant OTUs to capture small differences in their entirety with an accuracy of 0.92.

Through a large-scale cross-cohort study, we found that the conclusions of numerous previous 16S rRNA sequencing analyses were hasty. The argument for causality requires experiments with a logical framework that abides by Koch’s Law throughout, which is currently lacking in most 16S rRNA studies. A microbiome-wide association analysis is the first step in finding the members of all floras associated with a disease. Then, the disease-associated members are isolated and cultured into pure strains or member-defined compositions, which are inoculated into sterile animal models. Afterwards, the animals are placed under the appropriate environmental conditions to cause disease. Finally, immunological mechanisms are utilized to elucidate how these bacteria from the human body molecularly interact with the host to result in disease initiation. After this cycle, causality can be confirmed. The bacteria with proven causality and their active products can be employed as not only biomarkers for the diagnosis and early prediction but also as novel targets for disease prevention and treatment. It currently appears that only about 10% microbiota may afflict human health. Most of the oral and gut bacteria are background noise, which are virtually eliminated after the sample size is expanded in the present study. Disease-related bacteria cannot be simply found if researchers rely on various indexes of microbial diversity provided by databases and conduct classification and cutting-dimension analyses.

In addition, all five studies excluded factors (such as systemic disease and recent periodontal treatment) that assumed a significant role in confounding. And we also unveiled that the expanded sample size largely attenuated potential variations that could impact the accuracy of the results, such as oral hygiene status. As three of the five included researches did not disclose their specific clinical characteristics corresponding to the samples (including age, gender, BMI, and smoking), these clinical characteristics were not taken into account in the model. However, to mitigate the influence of these characteristics on the results, we evaluated the effects of available clinical characteristics on Rothia content. The regression analysis displayed that only the effects of age were significant (*p* < 0.01), and that the effects of gender, BMI, and smoking were not significant (*p* > 0.01). Similarly, the available results demonstrated that a range of clinical characteristics, represented by geography or ethnicity, could remarkably influence the ability of any classification models to diagnose T2DM, such as the higher Rothia sp. in US and Portugal populations (**Figure 1A**). Therefore, microbiological indicators should not be pursued to become a unified clinical standard for human beings but rather identify abnormal alterations under the microbiological characteristics of each specific population.

The diagnostic model provided by two of our included studies (in China) unraveled a substantial reduction in AUC when applied to another study (in the USA), which provided evidence for the salivary microbiological discrepancy in T2DM populations under different geographical regions. Interestingly, the study conducted by Almeida-Santos et al. has a relatively small sample size (n = 47) among the five studies. However, the microbial composition of this study was the most similar to the present study, especially the identical dominant bacteria at the phylum level (Almeida-Santos et al., 2021). The study also had the most homogeneous distribution in the PCoA, covering the quadrant, whilst the other four studies presented uneven distribution (**Figure 1B**). We strongly hypothesized that this was related to the mixed Caucasian and Yellow ancestry of Portugal, making its characteristics intermediate between those of the American and Chinese subjects.

On the other hand, the independent cohort validation has shown that salivary and subgingival microbial alterations are similar in patients with T2DM. We hypothesize that the unique microorganisms in saliva of T2DM patients are likely to originate from these eco-locations. However, our analysis unveiled that the microbial alterations characterized by saliva samples are extremely subtle and saliva samples might not be the best choice for identifying the microorganisms that could characterize T2DM patients. The oral cavity is classified into numerous different ecological sites, in which the bacteria communicate with each other through saliva, but their characteristics are totally variable. The eco-location and physicochemical environment of subgingival plaque and gingival sulcus are more specific than most of the other sites (Mark Welch et al., 2020). Therefore, the alterations may be similar in subgingival microorganisms, but are amplified. This suggests that subsequent researchers should prefer to take subgingival plaque as study subjects in order to complete oral microbiological studies related to periodontitis, although most studies have chosen to acquire saliva samples to represent the oral microbiome. Care should be taken when comparing or combining these studies to differentiate the sites sampled, such as gingival sulcus fluid and subgingival biofilm (Babaev et al., 2017; Demmer et al., 2019; Balmasova et al., 2021).

The correlation between DM and septicemia has been confirmed in several pieces of evidence (Yende and van der Poll, 2009; Schuetz et al., 2011), which explains the fact that an enhanced proportion of human pathogen septicemia functional genes is observed in the saliva of T2DM patients. The main reason for which T2DM has susceptibility to infection appears to be abnormalities of the host response, particularly of neutrophil chemotaxis, adhesion and intracellular killing, and defects that have been attributed to the effect of hyperglycemia (Koh et al., 2012). The importance of this discovery is that we added human pathogens septicemia for reconstructing the RF model as the 31st variable, yielding a recall of 0.93, which may contribute to the early prevention and monitoring of T2DM.

To the best of our knowledge, our research is the first large-sample analysis of oral microbiology in T2DM patients. We believe that as the sample size continues to expand, the salivary microbial diversity may become more similar between T2DM patients and healthy populations. Due to the unclear mechanisms of Rothia sp. in the pathogenic process of T2DM, it is not certain that its significant growth will be influenced by the larger sample size. This suggests that the positive results from prior studies are likely to be influenced by confounding factors. Multicenter clinical studies are still awaited to provide further evidence for this conjecture.

## Data Availability Statement

Publicly available datasets were analyzed in this study. This data can be found here: PRJNA561495 PRJNA601054 PRJNA609009 PRJNA679485 PRJNA664107.

## Author Contributions

CG and YG conceived and designed the project. CG downloaded, analyzed data, interpreted results, produced figures and drafted the manuscript. FC revised the manuscript. CG, YG, and FC all contributed to manuscript writing and editing. All authors read and approved the final manuscript.

## Funding

This work is supported by the National Science Foundation China (grant no. 81991501) and the open project of State Key Laboratory of Oral Diseases (grant no. SKLOD2021OF03).

## Conflict of Interest

The authors declare that the research was conducted in the absence of any commercial or financial relationships that could be construed as a potential conflict of interest.

## Publisher’s Note

All claims expressed in this article are solely those of the authors and do not necessarily represent those of their affiliated organizations, or those of the publisher, the editors and the reviewers. Any product that may be evaluated in this article, or claim that may be made by its manufacturer, is not guaranteed or endorsed by the publisher.
