# Supplementary figures and images for: Cross-Cohort Microbiome Analysis of Salivary Biomarkers in Patients With Type 2 Diabetes Mellitus
Source: Front Cell Infect Microbiol. 2022 Jan 25;12:816526. doi: 10.3389/fcimb.2022.816526 (PMC8821939; doi:10.3389/fcimb.2022.816526)

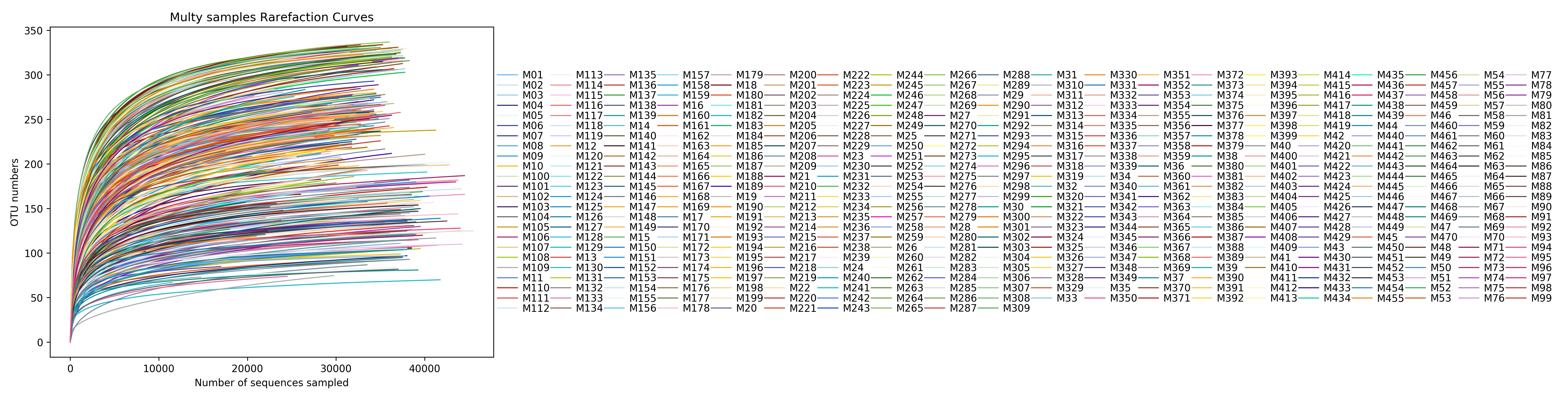

Supplement: Supplementary file 1 [file Image_1.jpeg]

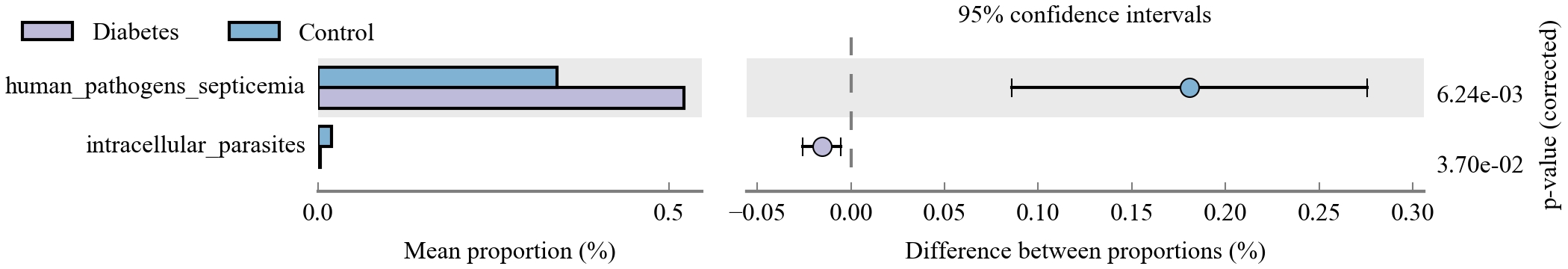

Supplement: Supplementary file 2 [file Image_2.jpeg]
